# Supplementary material for: Unpredictability of escape trajectory explains predator evasion ability and microhabitat preference of desert rodents
Source: Nat Commun. 2017 Sep 5;8:440. doi: 10.1038/s41467-017-00373-2 (PMC5585173; doi:10.1038/s41467-017-00373-2)
Supplement: Supplementary file 1 — Supplementary Information [file 41467_2017_373_MOESM1_ESM.pdf]

SI GUIDE

File Name: Supplementary Information
Description: Supplementary Figures, Supplementary Tables, Supplementary Notes Supplementary Methods and Supplementary References.

Supplementary Movies

File Name: Supplementary Movie 1
Description: Jaculus jaculus displaying the hopping gait. Hopping was associated with the greatest values of acceleration and deceleration, and was preferentially associated with stopping. Video is played back at 1/15x speed.

File Name: Supplementary Movie 2
Description: Jaculus jaculus displaying the skipping gait. Skipping exhibited the most symmetrical variation in mean acceleration across the broadest range of speeds. Video is played back at 1/15x speed.

File Name: Supplementary Movie 3
Description: Jaculus jaculus displaying the running gait. Running showed the least variation in mean acceleration, and was used primarily at lower speeds. Video is played back at 1/15x speed.

File Name: Supplementary Movie 4
Description: Jaculus jaculus displaying a gait transition. Frequent transitions between gaits that exhibit distinct dynamic functions may increase the jerboa's capacity for maneuverability. Video is played back at 1/15x speed.

File Name: Supplementary Movie 5
Description: Video clip of quadrupedal Meriones sp. locomotion in response to simulated predation. These videos were also used to perform the OpenField Anxiety test.

File Name: Supplementary Movie 6
Description: Video clip of bipedal Allactaga elater locomotion in response to simulated predation. These videos were also used to perform the OpenField Anxiety test.

File Name: Supplementary Movie 7
Description: Video clip of quadrupedal Meriones unguiculatus locomotion during Light-Dark Box Exploration test in a laboratory. Video is played back at 10x speed.

File Name: Supplementary Movie 8
Description: Video clip of bipedal Jaculus jaculus locomotion during Light-Dark Box Exploration test in a laboratory. Video is played back at 10x speed.

File Name: Peer Review File
Description:

Supplementary Figures

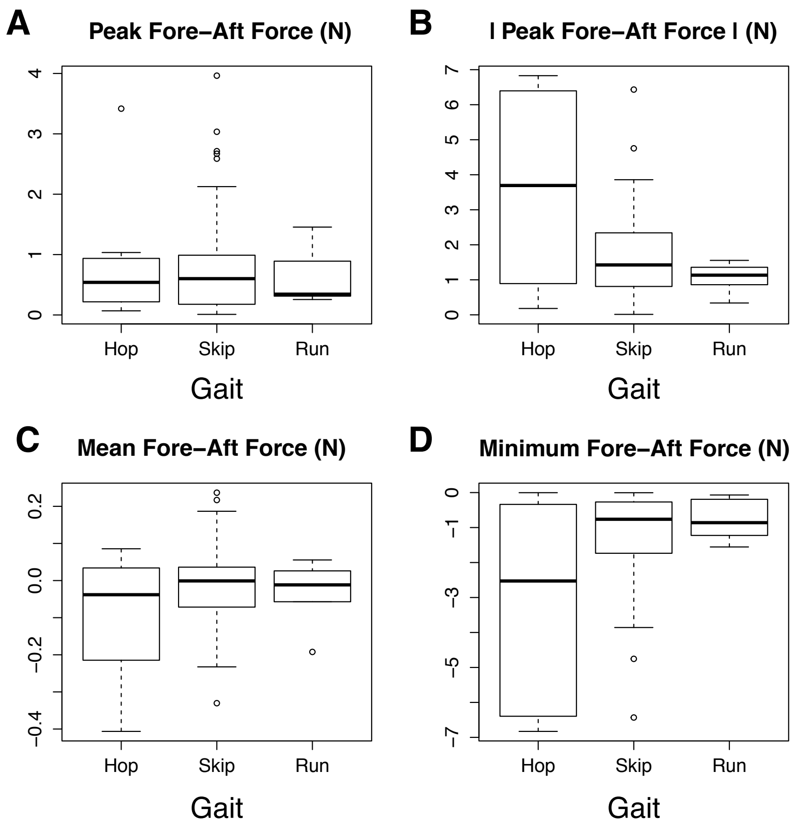

Supplementary Figure 1: Components of fore-aft force sorted by gait. A) Peak fore-aft force did not differ significantly between gaits ($F_{2,77} = 0.22$, $p = 0.801$, one-way ANOVA), B) Peak magnitude of vertical force was highly significant, indicating that negative fore-aft force differs significantly between gait ($F_{2,77} = 9.32$, $p = 0.000237$, one-way ANOVA), C) Mean fore-aft force did not differ significantly between gait ($F_{2,77} = 2.06$, $p = 0.135$, one-way ANOVA), D) Minimum fore-aft force was highly significant ($F_{2,77} = 7.10$, $p = 0.00148$, one-way ANOVA), though not as significant as the magnitude of vertical force, indicating that both large negative and positive fore-aft forces contribute to the significant differences between gait. The boxes span the interquartile range, the bold line represents the median, the whiskers extend to 1.5 times the interquartile range, and the open circles show outlier values outside of the whiskers.

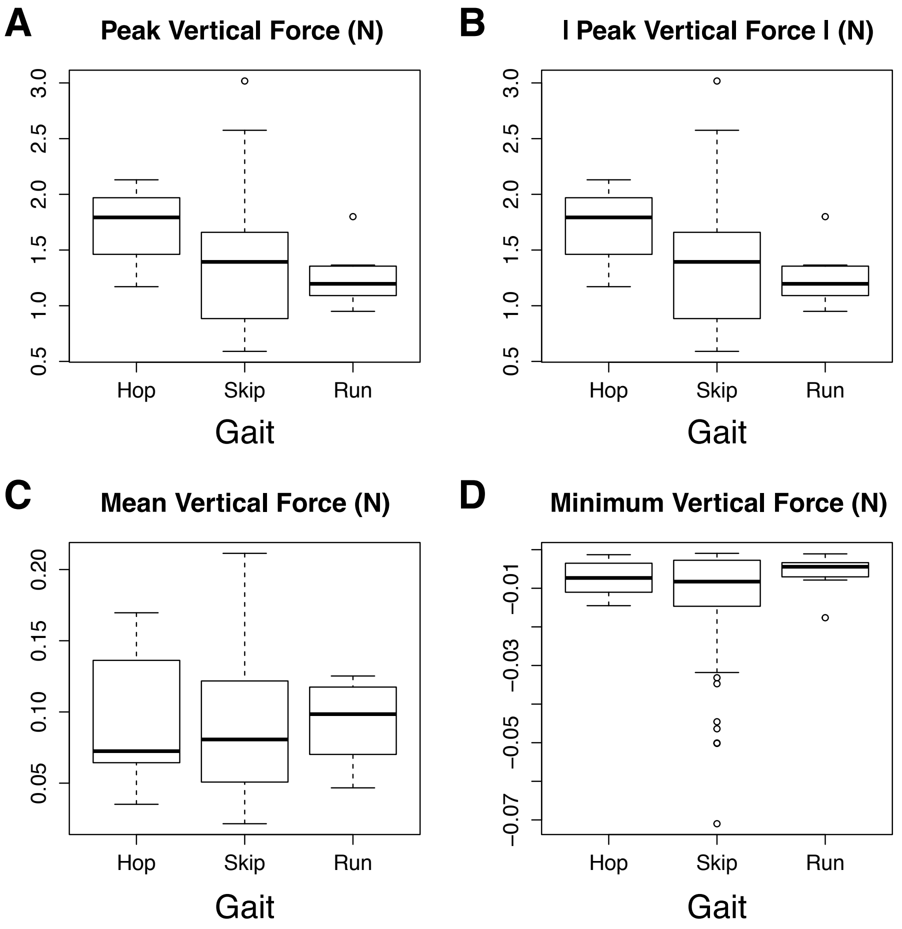

Supplementary Figure 2: Components of vertical force sorted by gait. A) Peak vertical force trended towards significantly different between gaits ($F_{2,77} = 2.51$, $p = 0.0876$, one-way ANOVA), B) Peak magnitude of vertical force was exactly the same, indicating that magnitude of positive vertical force was greater than the magnitude of negative vertical force ($F_{2,77} = 2.51$, $p = 0.0876$, one-way ANOVA), C) Mean vertical force was not significantly different between gaits ($F_{2,77} = 0.003$, $p = 0.997$, one-way ANOVA), D) Minimum vertical force was near zero, indicating accurate balancing of bridges and minimal ringing, and was not significantly different between gaits ($F_{2,77} = 1.43$, $p = 0.246$, one-way ANOVA). The boxes span the interquartile range, the bold line represents the median, the whiskers extend to 1.5 times the interquartile range, and the open circles show outlier values outside of the whiskers.

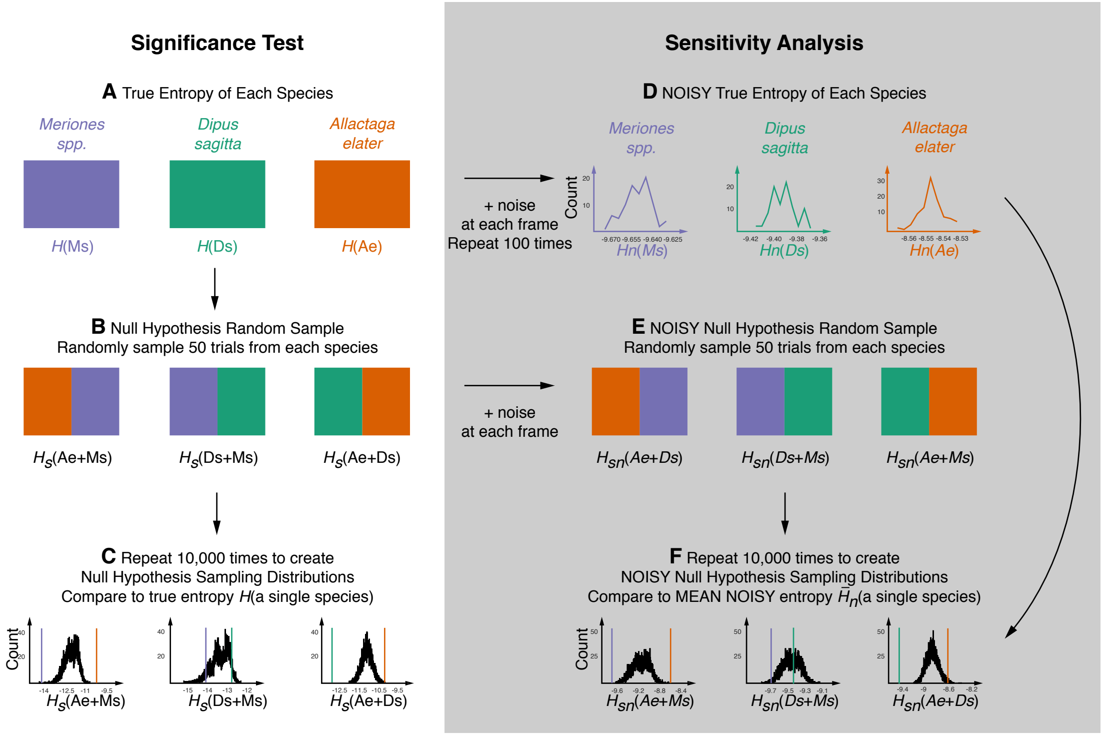

Supplementary Figure 3: Flowchart describing the test for significant difference between species (A, B, and C) and the test for sensitivity to tracking error (D, E, and F in gray). A) The true entropy of each species, H(species) was calculated from all of the trajectory data for a given species as described in the Methods and Supplementary Methods. B) Sample datasets were created to test the null hypothesis that the species are not significantly different from each other by randomly sampling 50 trials from each unique species pair. The entropy of this combined dataset was measured, H_s(species pair). C) The sample datasets were randomly recreated 10,000 times, and the entropy of each sample dataset was stored in a sampling distribution. The true entropy, H(species), for each species compared was then plotted on the sampling distribution. The proportion of the sampling distribution lying outside of the true entropy for each species (colored vertical lines in C), divided by the total number of sample datasets (10,000), provided the p-value for each species in the comparison. To understand how tracking error would affect the relationship between the species trajectory entropies, we added random error and repeated the significance test. D) First, we calculated the noisy entropy H_n(species) for a single species by randomly sampling error from a Gaussian distribution and adding error to each frame of data. We repeated this 100 times to provide the sampling distribution for the noisy entropy of a single species, and calculated the mean, $\bar{H}_n$(species). E) We repeated the creation of the sample datasets for each species pair as in B), added randomly sampled error to each frame of data, and measured the entropy of the noisy sample dataset, $H_s n$(species pair). F) We repeated the creation of noisy sample datasets 10,000 times to generate a noisy sampling distribution, and compared the mass of the distribution to the mean noisy entropy of a single species, $\bar{H}_n$(species) from D), to determine the p-value for each species in the comparison. Note, the distributions in C, D, and F are to scale.

Supplementary Table

	% Exposed	% Risk Assess	% Out to In	% Risk to In
Species+Individual (Nested)	***,*	-,-	***,*	***,-
Species*Trial Number (Mixed)	**,.,-	-,-,*	.,-,-	***,-,-
Species*Capture Time (Mixed)	*,-,-	-,-,-	.,-,-	***,-,-
Species*Box Location (Mixed)	*,-,-	-,-,-	.,-,-	***,-,-

Supplementary Table 1: A summary table showing the level of significance for the effect of each independent variable on the behavior of animals in the Light-Dark Box Exploration test using 44 one-way ANOVAs. The dependent variables are the column headings, and the independent variables and the type of model used are the row headings. Symbols indicate first the effect of the species, then the effect of the additional variable, and, if present, the mixed effects of species and the additional variable. - indicates $p > 0.1$, . indicates $0.1 > p > 0.05$, * indicates $0.05 > p > 0.01$, ** indicates $0.01 > p > 0.001$, *** indicates $0.001 > p > 0$. All models were designed prior to performing the experiment.

Supplementary Notes

Laboratory-Based Light-Dark Box Exploration Test

Species was the main determinant of microhabitat preference (Nested ANOVA) for the percentage of time spent in the exposed area and the number of times the animal returned to the box after a period of risk assessment (Supplementary Table 1). Variations specific to each individual and trial number, or acclimation to the experiment, were marginally significant determinants of behavior. We found that the box location, time it took to capture the animal, and the time in each animal's light cycle the experiment was performed had no measurable effect on the difference between species ($\alpha = 0.05$) on percentage of time spent in the exposed area, the number of times the animal went into the box, or the number of times the animal returned to the box after a period of risk assessment (Mixed-effects ANOVA). The percentage of time in risk-assessment only varied significantly with the combined effect of species and trial number.

Supplementary Methods

Note on Mathematical Proofs for Continuous Entropy Calculation

In this section we elaborate on the optimization problem (6) that seeks to find a continuous probability density function that matches the trajectory data from a given species. We use optimization to ensure that the distribution simultaneously maximizes the differential entropy while matching the empirical moments of the trajectory data. Note, we make substantial use of measure theory, and the unfamiliar reader may wish to consult Folland[1] for an introduction.

Notation

Here we introduce notation to describe polynomial equations, using the dummy variables y and p for the purpose of generalization.

$\mathbb{R}^{n\times m}$ denotes the set of matrices that have n rows and m columns. Given $A \in \mathbb{R}^{n\times m}$, let A_{ij} denote the (i,j)-th component of A. By $\mathbb{N}$ we denote the nonnegative integers and let $\mathbb{N}^n_k$ refer to those $\boldsymbol{\alpha} \in \mathbb{N}^n$ with $|\boldsymbol{\alpha}| = \sum_{i=1}^n \boldsymbol{\alpha}_i \leq k$. In other words, $\mathbb{N}^n_k$ represents the n-dimensional non-negative integers whose sum is less than or equal to k.

Every polynomial on $\mathbb{R}^n$ with $\mathbf{x} = (\mathbf{x}_1, \ldots, \mathbf{x}_n)$, can be expanded in the monomial bases via:

$$p(\mathbf{x}) = \sum_{\boldsymbol{\alpha}\in\mathbb{N}^n} \mathbf{p}_{\boldsymbol{\alpha}} \mathbf{x}^{\boldsymbol{\alpha}} \tag{1}$$

where $\mathbf{x}^{\boldsymbol{\alpha}}$ denotes $\mathbf{x}_1^{\boldsymbol{\alpha}_1} \cdots \mathbf{x}_n^{\boldsymbol{\alpha}_n}$ and $(\mathbf{p}_{\boldsymbol{\alpha}})_{\boldsymbol{\alpha}\in\mathbb{N}^n}$ is the vector of coefficients of p. For example, the polynomial $p(\mathbf{x}_1,\mathbf{x}_2) = 1 + 2\mathbf{x}_1\mathbf{x}_2 + 7\mathbf{x}_1^2$ can also be written in the form $p(\mathbf{x}_1,\mathbf{x}_2) = \sum_{\alpha\in\mathbb{N}^2} \mathbf{p}_{\boldsymbol{\alpha}_1,\boldsymbol{\alpha}_2} \mathbf{x}_1^{\boldsymbol{\alpha}_1}\mathbf{x}_2^{\boldsymbol{\alpha}_2}$, where $\boldsymbol{\alpha}$ represents the exponent of each variable, and $\mathbf{p}_{\boldsymbol{\alpha}_1,\boldsymbol{\alpha}_2}$ are the coefficients of the variables of each power. In this example, $\mathbf{p}_{0,0} = 1$, $\mathbf{p}_{1,1} = 2$, $\mathbf{p}_{2,0} = 7$, and $\mathbf{p}_{\boldsymbol{\alpha}_1,\boldsymbol{\alpha}_2} = 0$ for every other $\boldsymbol{\alpha}_1$ and $\boldsymbol{\alpha}_2$.

Optimization Problem (6)

Solving optimization problem (6) requires finding an object defined on a continuum. Fortunately, one can prove (refer to Lemma 1) that this optimization problem has a solution parameterized by

a finite-dimensional vector. That is, this optimization problem has a solution of the following form:

$$f^*(\boldsymbol{\lambda}^*, \mathbf{x}) = \exp(\sum_{\boldsymbol{\alpha} \in \mathbb{N}^3_{2k}} \boldsymbol{\lambda}^*_{\boldsymbol{\alpha}} \mathbf{x}^{\boldsymbol{\alpha}}), \tag{2}$$

where $\boldsymbol{\lambda}^*$ is a real-valued vector of size $\binom{3+2k}{2k}$, that is 3+2k choose 2k, whose $\boldsymbol{\alpha}$-component is denoted by $\boldsymbol{\lambda}^*_{\boldsymbol{\alpha}}$ for each $\boldsymbol{\alpha} \in \mathbb{N}^3_{2k}$. Note that asterisks indicate an optimal value for the accompanying variable. $\boldsymbol{\lambda}^*$ is a vector of coefficients of the polynomial solution to problem (6). By searching for the dimensional vector $\boldsymbol{\lambda}^*$ that maximizes the differential entropy while matching the empirical moments, one has solved for the continuous probability distribution that best describes the empirical data while making the fewest assumptions about the data.

Lemma 1 *There exists a finite vector* $\boldsymbol{\lambda}^* = (\boldsymbol{\lambda}^*_{\boldsymbol{\alpha}})_{|\boldsymbol{\alpha}| \leq 2k} \in \mathbb{R}^{\binom{3+2k}{2k}}$ *such that:*

$$f^*(\boldsymbol{\lambda}^*, \mathbf{x}) = \exp(\sum_{\boldsymbol{\alpha} \in \mathbb{N}^3_{2k}} \boldsymbol{\lambda}^*_{\boldsymbol{\alpha}} \mathbf{x}^{\boldsymbol{\alpha}}), \tag{3}$$

is a solution to Problem (6).

Proof: Suppose $g \in L^1\left([-1,1]^3\right)$ has moments with order less than or equal to $2k$ that are identical to f^* which is given by Equation (3):

$$\begin{aligned}
\boldsymbol{H}[g] &= -\int\limits_{[-1,1]^3} g(\mathbf{x}) \ln\left(g(\mathbf{x}) \frac{f^*(\boldsymbol{\lambda}^*, \mathbf{x})}{f^*(\boldsymbol{\lambda}^*, \mathbf{x})}\right) d\mathbf{x} && (4)\\
&= -KL(g||f^*) - \int\limits_{[-1,1]^3} g(\mathbf{x}) \ln f^*(\boldsymbol{\lambda}^*, \mathbf{x}) d\mathbf{x} && (5)\\
&\leq -\int\limits_{[-1,1]^3} g(\mathbf{x}) \left(\sum_{\boldsymbol{\alpha} \in \mathbb{N}^3_{2k}} \boldsymbol{\lambda}^*_{\boldsymbol{\alpha}} \mathbf{x}^{\boldsymbol{\alpha}}\right) d\mathbf{x} && (6)\\
&= -\sum_{\boldsymbol{\alpha} \in \mathbb{N}^3_{2k}} \boldsymbol{\lambda}^*_{\boldsymbol{\alpha}} \mathbf{m}_{\boldsymbol{\alpha}} && (7)\\
&= -\int\limits_{[-1,1]^3} f^*(\boldsymbol{\lambda}^*, \mathbf{x}) \left(\sum_{\boldsymbol{\alpha} \in \mathbb{N}^3_{2k}} \lambda^*_{\boldsymbol{\alpha}} \mathbf{x}^{\boldsymbol{\alpha}}\right) d\mathbf{x} && (8)\\
&= -\int\limits_{[-1,1]^3} f^*(\boldsymbol{\lambda}^*, \mathbf{x}) \ln f^*(\boldsymbol{\lambda}^*, \mathbf{x}) d\mathbf{x} && (9)\\
&= H[f], && (10)
\end{aligned}$$

where in the second line KL denotes the Kullback-Leibler Divergence,[2] which is always nonnegative, and the fourth line follows since g and f have identical moments for all moments with order less than or equal to $2k$. □

As a result of Lemma 1 and by applying the Legendre-Fenchel Transformation, one can prove that solving Equation (6) reduces to solving the concave finite-dimensional optimization problem:[3]

$$\sup_{\boldsymbol{\lambda} \in \mathbb{R}^{\binom{3+2k}{2k}}} \left\{ \sum_{\boldsymbol{\alpha} \in \mathbb{N}^3_{2k}} \widehat{\mathbf{m}}_{\boldsymbol{\alpha}} \boldsymbol{\lambda}_{\boldsymbol{\alpha}} - f_d(\boldsymbol{\lambda}) \right\}, \tag{11}$$

where $f_d : \mathbb{R}^{\binom{3+2k}{2k}} \to \mathbb{R}$ is defined as:

$$f_d(\boldsymbol{\lambda}) = \int\limits_{[-1,1]^3} f(\boldsymbol{\lambda}, \mathbf{x}) d\mathbf{x}. \tag{12}$$

One can efficiently solve the optimization problem in Equation (11) by relying on Newton's method as described in Algorithm 1. Applying this method requires the second derivative of f_d which is called the Hessian. To compute this Hessian, let us denote the $\boldsymbol{\alpha} \in \mathbb{N}^3$ moment of $f(\boldsymbol{\lambda}, \mathbf{x})$ as $\widetilde{\mathbf{m}}_{\boldsymbol{\alpha}}(\boldsymbol{\lambda})$ where:

$$\widetilde{\mathbf{m}}_\alpha(\boldsymbol{\lambda}) = \int\limits_{[-1,1]^3} \mathbf{x}^\alpha f(\boldsymbol{\lambda}, \mathbf{x}) d\mathbf{x}. \tag{13}$$

Given $\boldsymbol{\lambda} \in \mathbb{R}^{\binom{3+2k}{2k}}$ one can compute $\widetilde{\mathbf{m}}_{\boldsymbol{\alpha}}(\boldsymbol{\lambda})$ using numerical integration. Given this definition, one can compute the Hessian of f_d according to the following Lemma:

Lemma 2 *The Hessian of f_d which is denoted by $\nabla^2 f_d : \mathbb{R}^{\binom{3+2k}{2k}} \to \mathbb{R}^{\binom{3+2k}{2k} \times \binom{3+2k}{2k}}$ is given by:*

$$\nabla^2 f_d(\boldsymbol{\lambda})_{\mathbf{j},\mathbf{k}} = \int\limits_{[-1,1]^3} x^{\mathbf{j}+\mathbf{k}} f(\boldsymbol{\lambda}, \mathbf{x}) d\mathbf{x} = \widetilde{\mathbf{m}}_{\mathbf{j}+\mathbf{k}}(\boldsymbol{\lambda}) \tag{14}$$

for all $\mathbf{j}, \mathbf{k} \in \mathbb{N}^3_{2k}$.

Proof: This follows directly from the Fundamental Theorem of Calculus. □

Require: $\boldsymbol{\lambda}^0 \in \mathbb{R}^{\binom{3+2k}{2k}}$ and $\epsilon \in (0, \infty)$.
  Set $\boldsymbol{\lambda}^1 = \boldsymbol{\lambda}^0 + \left[\nabla^2 f_d(\boldsymbol{\lambda}^0)\right]^{-1} (\widehat{\mathbf{m}} - \widetilde{\mathbf{m}}(\boldsymbol{\lambda}^0))$
  Set $j = 1$.
  while $\|\boldsymbol{\lambda}^{j-1} - \boldsymbol{\lambda}^j\|_2 \leq \epsilon$ **do**
    Set $\boldsymbol{\lambda}^{j+1} = \boldsymbol{\lambda}^j + \left[\nabla^2 f_d(\boldsymbol{\lambda}^j)\right]^{-1} (\widehat{\mathbf{m}} - \widetilde{\mathbf{m}}(\boldsymbol{\lambda}^j))$
    Replace j by $j + 1$.
  end while
  return $\boldsymbol{\lambda}^j$.

Algorithm 1: Newton's Method for Computing the Maximum Entropy Distribution

Entropy Significance Test

To determine whether there are significant differences in entropy between species pairs, we compared the entropy computed for each species (H(species) in Supplementary Fig. 3 A) to the entropy of a dataset containing trials from an equal number of trials from both species (i.e. *A. elater* vs *D. sagitta*, *A. elater* vs. *Meriones sp.*, and *D. sagitta* Vs. *Meriones sp.*). We tested the null hypothesis that there is no difference between the trajectory entropy of the two species.

For each unique pair of species, we computed the entropy of a random sample of 50 trials from the continuous probability distribution of each species, (100 trials sampled total, H_s(species pair) in Supplementary Fig. 3 B). We repeated this process 10,000 times to generate a sampling distribution (Supplementary Fig. 3 C). For each pair of species we determined the proportion of the sampling distribution that is either greater than or less than the true entropy value of

each species in the comparison (colored lines in Supplementary Fig. 3 C represent the H(species) values from A). This outlying proportion divided by the total number of values (10,000) represents the p-value, or how significantly the data reject the null hypothesis.

Entropy Divergence Sensitivity To Tracking Error

We performed an analysis to determine whether imprecise tracking of the animal center of mass would affect the relationship between the entropy of trajectories performed by each species. All three rodent species are approximately the same size — 8 cm long (x-axis), 4 cm wide (y-axis), and 4 cm high (z-axis) — and all videos were tracked by the same individual, so we assumed that the error in tracking the center of mass was similar for all videos. Error was modeled as random samples from a Gaussian distribution centered at 0 for each axis, with a standard deviation of 1/8 the length of the body in each axis (1 cm for the x-axis, and 0.5 cm for the y- and z-axes).

To test whether our results were robust to this tracking error, we re-ran the significance test. We randomly sampled an equal number of trials from each of two species to test for comparisons between them (as in Section above), then added error (randomly sampled from the error distribution) to each frame, measured the entropy of this noisy random sample (H_{sn}(species pair) in Supplementary Fig. 3 E), and repeated 10,000 times to generate a noisy sampling distribution (Supplementary Fig. 3 F). To determine the 'true' value for each species while incorporating error, we added a randomly sampled error value to each frame from each species, measured the entropy of the noisy single species distribution, and repeated this 100 times (H_n(species) in Supplementary Fig. 3 D). To estimate the significance between species in the two species comparisons, we measured the proportion of the sampling distribution lying outside of the mean noisy single species entropy ($\bar{H}_n$(species) in Supplementary Fig. 3 D, and the colored vertical lines in Supplementary Fig. 3 F).

We note that the introduction of random error should increase the entropy of a distribution. For example, if the animal is stationary for several frames, the error would induce a small amount of movement in the data. Consistent with this, we found that the entropy of each single species distribution increased with the addition of error ($\bar{H}_n(A.\ elater)$ = -8.5422, $\bar{H}_n(D.\ sagitta)$ = -9.3867, and $\bar{H}_n(Meriones\ sp.)$ = -9.6453). Despite this change in the entropy of each species, the overall pattern of significance in the differences between species was preserved (Supplementary Fig. 3 E). In the noisy significance test between *A. elater* and *D. sagitta*, $p_n(A.\ elater) = 0.0081$ and $p_n(D.\ sagitta) = 0$, strongly significantly different. In the noisy significance test between *A. elater* and *Meriones sp.*, $p_n(A.\ elater) = 0$ and $p_n(Meriones\ sp.) = 0$, also strongly significantly different. In the noisy significance test between *D. sagitta* and *Meriones sp.*, $p_n(D.\ sagitta)$ = 0.4378 and $p_n(Meriones\ sp.)$ = 0.0087, indicating that *Meriones sp.* is significantly more predictable than a combined sampling distribution of noisy *D. sagitta* and noisy *Meriones sp.* trajectories, but *D. sagitta* is not significantly more unpredictable than this combined noisy sampling distribution.

Supplementary References

[1] Folland, G B (2013). *Real analysis: modern techniques and their applications.* Wiley.

[2] Cover, T M, Thomas, J A (2012). *Elements of information theory.* Wiley.

[3] Lasserre, J B (2009). *Moments, positive polynomials and their applications (Vol. 1).* World Scientific.
